# State of the Art on Developments of (Bio)Sensors and Analytical Methods for Rifamycin Antibiotics Determination

**DOI:** 10.3390/s23020976

**Published:** 2023-01-14

**Authors:** Hassan Noor, Iulia Gabriela David, Maria Lorena Jinga, Dana Elena Popa, Mihaela Buleandra, Emilia Elena Iorgulescu, Adela Magdalena Ciobanu

**Affiliations:** 1 Department of Surgery, Faculty of Medicine, “Lucian Blaga” University Sibiu, Lucian Blaga Street 25, 550169 Sibiu, Romania; 2 Department of Analytical Chemistry and Physical Chemistry, Faculty of Chemistry, University of Bucharest, Panduri Av. 90-92, District 5, 050663 Bucharest, Romania; 3 Department of Psychiatry “Prof. Dr. Al. Obregia” Clinical Hospital of Psychiatry, Berceni Av. 10, District 4, 041914 Bucharest, Romania; 4 Discipline of Psychiatry, Neurosciences Department, Faculty of Medicine, “Carol Davila” University of Medicine and Pharmacy, Dionisie Lupu Street 37, 020021 Bucharest, Romania

**Keywords:** rifamycin, rifampicin, (bio)sensor, electrochemical, spectrometric, fluorescence, chemiluminescence

## Abstract

This review summarizes the literature data reported from 2000 up to the present on the development of various electrochemical (voltammetric, amperometric, potentiometric and photoelectrochemical), optical (UV-Vis and IR) and luminescence (chemiluminescence and fluorescence) methods and the corresponding sensors for rifamycin antibiotics analysis. The discussion is focused mainly on the foremost compound of this class of macrocyclic drugs, namely rifampicin (RIF), which is a first-line antituberculosis agent derived from rifampicin SV (RSV). RIF and RSV also have excellent therapeutic action in the treatment of other bacterial infectious diseases. Due to the side-effects (e.g., prevalence of drug-resistant bacteria, hepatotoxicity) of long-term RIF intake, drug monitoring in patients is of real importance in establishing the optimum RIF dose, and therefore, reliable, rapid and simple methods of analysis are required. Based on the studies published on this topic in the last two decades, the sensing principles, some examples of sensors preparation procedures, as well as the performance characteristics (linear range, limits of detection and quantification) of analytical methods for RIF determination, are compared and correlated, critically emphasizing their benefits and limitations. Examples of spectrometric and electrochemical investigations of RIF interaction with biologically important molecules are also presented.

## 1. Introduction

Antibiotics are natural, semi-synthetic or synthetic molecules with the ability to kill or stop the growth of various types of microorganisms, being used as antimicrobial agents. Since the accidental discovery of penicillin in 1928 by Sir Alexander Flemming, many other antibiotics belonging to different classes (e.g., tetracyclines, sulfonamides, amphenicols, aminoglycosides, β-lactams, etc.) have been determined based on their chemical structure or action mechanisms. They have been produced and have brought improvement to the clinical field [1] due to their employment in the treatment of bacterial infectious diseases in humans, animals and even plants [2]. However, the increased use of antibiotics, sometimes uncontrolled, has resulted in the malfunction of organs, damage of nervous, renal or blood systems and in the enhanced development of antibiotic resistance. Therefore, personalized antibiotherapy is highly recommended and this can be realized by TDM, applying point-of-care testing, which can be easily performed with specific biosensors. A microfluidic electrochemical biosensor was described for monitoring the β-lactam antibiotics levels in different biological matrices (whole blood, plasma, urine, saliva and exhaled breath condensate) [3]. Some veterinary antibiotics have not only been used for prophylactic or treatment purposes, but they have also been gradually added to feed in order to improve the growth rate of livestock and poultry, and thus, can accumulate in agricultural and husbandry products, which are consumed by humans (e.g., meat, eggs, milk, honey and fish) [2,4]. Antibiotics are excreted from organisms into the environment either as the parent drug or as its metabolites. Other ways by which antibiotics enter into the waterways and ecosystems are aquaculture medication and industrial wastewaters [5]. Because they are non-biodegradable and possess low water solubility, they accumulate and remain in the environment as persistent contaminants, becoming hazardous for human health and ecosystems [2]. Considering all of these features, it is obvious that the continuous development of cheap, fast and reliable sensors and methods that allow for the detection and quantification of antibiotics from different matrices is necessary. This fact is emphasized by the already existing reviews regarding antibiotics analysis using electrochemical sensors [6], with some of them being focused on a given class of antibiotics like amphenicols [7] or aminoglycosides [8,9], or on various types of modifiers like graphene [5,10], MIPs [1], nanomaterials [11,12], metal organic frameworks [2] or fluorescent sensors based on luminescent metal-organic frameworks [13]. Two recent reviews focused on biosensors relying on various biorecognition elements and nanomaterials applied to antibiotic detection in food matrices [4,14]. 

The importance of biosensing technology in the development of either new antibiotics or various carriers for their delivery must be considered. Examples of biosensors with various detection modes applied for drug discovery and analysis, in high throughput screening, real-time and online monitoring of (bio)chemical processes for pharmaceutical production and quality control of the final products, were discussed by Finny, Cheng and Andreescu [15].

Over the years, rifamycins has proved to be an important antibiotic class, indispensable in TB therapy, with RIF being the first-line medication in the treatment of this worldwide spread, often lethal disease. Despite the fact that there have been several reviews regarding antibiotics analysis, these do not address rifamycins. Further to their therapeutic importance, rifamycins have disadvantages, so the administrated dose must be controlled and optimized to minimize risks and to not be dangerous for either the ecosystem or for human beings. All of these aspects demonstrate the importance of various types of (bio)sensors and analytical methods development, thus allowing the sensitive and selective detection of these compounds, especially of RIF. Desai and Sash, in 2015, published a review discussing spectrophotometric, spectrofluorimetric and chromatographic methods reported between 1997 and 2014 for RIF determination [16]. Thapliyal et al. [17] provided an overview of the papers published from 2006 to 2015 regarding the electrochemical methods devised for the detection of antitubercular drugs. The evolution of analytical separation (high performance and ultra-high performance liquid chromatography, thin layer chromatography, capillary electrophoresis) and spectroscopic techniques (UV, NIR, FTIR, Raman) associated with a chemometric approach for the simultaneous determination of RIF, ISN, PYR and ETB in fixed-dose combination anti-tuberculosis pharmaceutical formulations was discussed by Oliveira et al. [18]. In 2019, Farokhi-Fard et al. [19] mentioned RIF and ISN electroanalysis at bare electrodes and offered a more detailed discussion on different nanomaterial electrode modifiers. In the most recent review regarding instrumental analytical methods reported for RIF quantification dates from 2021, around 70% of the paper referred to chromatographic and hyphenated techniques, and briefly discussed spectrometric techniques, among others [20]. As will be highlighted in this review, our recent literature survey revealed that there is also a huge interest in the electrochemical (bio)sensors and methods for RIF analysis. However, recent information regarding this issue has not been exhaustively collected and summarized over the last few years. The present work offers an overview and critically discusses the literature data reported from 2000 to today on the development of various electrochemical (voltammetric, amperometric, potentiometric and photoelectrochemical), optical (UV-Vis and IR) and luminescence (chemiluminescence and fluorescence) methods, as well as the corresponding (bio)sensors for the analysis of rifamycin antibiotics, with emphasis on RIF, in pharmaceutical formulations, biological fluids and environmental samples. Some recent findings in RIF delivery systems are concisely presented together with the analytical procedures employed for in vitro RIF release monitoring during their preparation. Understanding the interaction between drugs and other species that may exist in the human body allows for an understanding of drug pharmacokinetics, their mode of action and the conditions in which their efficiency can be improved. Therefore, a special section of this review is dedicated to the electrochemical and spectrometric investigations of RIF interaction with biologically important molecules.

## 2. Rifamycins—Properties and Clinical Treatment

**Figure 1:**
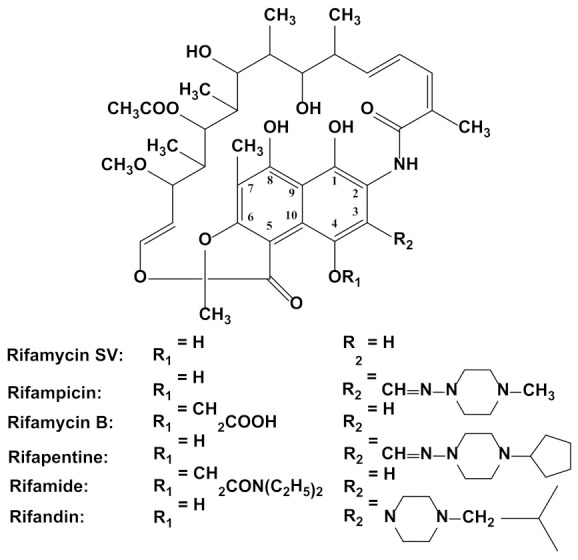
Chemical structure of the most common rifamycins.

Rifamycins are natural or semisynthetic macrocyclic antibacterial agents active against a large variety of organisms, including bacteria and eukaryotes [21]. They belong to the ansamycin family, the name of which originates from the Latin word “ansa”, meaning “handle”, and is due to their basket-like molecular architecture [22,23] (Figure 1). It is interesting to note that the name “rifamycin” refers to the popular French noir crime film from 1955, *Rififi* [24]. The compounds of this class have an “ansa structure”, constituted by a core formed from condensed naphthalene and furan rings [25] and a highly substituted aliphatic chain connecting the two nonadjacent positions of the naphtohydroquinone ring [21,26,27], which is also the chromophore responsible for their red-orange color [28,29]. The molecular structures of the various rifamycins differ by the type and the position of the substituents on the aromatic ring; the most common representatives of this antibiotic class are shown in Figure 1 [26,30]. These molecules have different functional groups (e.g., −OH in both the aliphatic bridge and on the aromatic rings, amine, amide, furanone, methoxy and acetyl) [31], thus being generated chiral centers that make the compounds optically active [26]. The presence of the hydroxyl groups confers them with antioxidant properties and makes them adequate for radicalic polymerization reactions [29].

A mixture of rifamycins A, B, C, D and E was first obtained in 1957 from a strain called, at that time, *Streptomyces mediterranei*, known today as *Amycolatopsis rifamycinica*. Rifamycins are unstable during the purification process, except for inactive rifamycin B, which is rapidly oxidized and hydrolyzed into the active rifamycin S. Chemical modification of rifamycin S generated RSV, the first ansamycin clinically used and one of the most important representative drugs of this class, in addition to RIF [32]. The rifamycins group also includes rifapentine (less employed due to its hepatotoxicity), rifabutin (applied as treatment for patients who do not tolerate RIF) [33], rifamide (limited to intravenous use), rifalazil (treats persistent chlamydia infections) and rifaximin (used for the prevention of travelers’ diarrhea) [32].

The most important representative of rifamycins is the hydrazone of a rifamycin B derivative with N-amino-N’-methylpiperazine [24], namely (3-[(4-methyl-1-piperazinyl) imino] methyl rifamycin, called rifampicin (RIF) (Figure 1). It is a semi-synthetic ansamycin, chemically derived from the natural rifamycin B via rifamycin S and RSV, respectively [32,34]. RIF, also known as rifaldazine or rifampin, or under various commercial names like Rifadin, Rimactan, Rifoldine, etc. [35], was discovered in 1965 [19] and approved for clinical use by the United States Food and Drug Administration in 1971 [24]. 

RIF is an odorless red crystalline powder. It is hydrophobic and very slightly soluble in water, acetone, alcohol and ether, and it is soluble in methanol and ethyl acetate, and easily soluble in chloroform [36,37]. Based on its reduced, pH-dependent water solubility (2.50 mg/mL at 25 °C) and highly permeability, RIF is classified as a Class II drug, according to the Biopharmaceutics Classification System [38,39]. RIF presents seven crystalline forms. The metastable polymorph II is used in commercialized pharmaceutical preparations because it has the highest water solubility [39].

From a chemical point of view, RIF is a hydroquinone derivative bearing three phenolic −OH groups, which can be chemically or electrochemically oxidized [40]. Due to the acidic hydroxyl group from position 4 of the aromatic structure (pK_a1_ = 1.70) and of the basic 3-piperazine nitrogen (pK_a2_ = 7.90), RIF exhibits an amphoteric character and, depending on the pH, it can exist as a zwitterion [19]. RIF is unstable and its degradation depends on the pH, temperature and storage period. In acidic medium, it is hydrolyzed to 3-formyl-RSV and 1-amino-4-methylpiperazine, and, in alkaline conditions, it autoxidized, the main degradation product being the inactive RIF quinone [41]. 

RIF is a broad spectrum antibiotic with effective action against Gram-positive and some Gram-negative bacterial strains [25,30,41,42,43]. The hydroxyl groups of RIF form hydrogen bonds with amino acid residues of the bacterial DNA-dependent RNA polymerase, resulting in a stable drug-enzyme complex, inhibiting the initiation of RNA synthesis and stopping bacterial growth [28,44].

RIF was clinically introduced to fight TB [45] and it is presently still the main medicine used to prevent or treat this ailment [33], which is considered to be in the top ten of the world’s fatal diseases and a leading cause of death in HIV patients [39] (in 2021 there were 1.6 million deaths from TB, with 187,000 of these among HIV-positive patients). The WHO Global Tuberculosis Report 2022 [46] estimated that, in 2021, the worldwide number of new TB cases (10.6 million) increased by 4.5% in comparison to 2020. Without treatment, the death rate of TB is about 50%. RIF is the most powerful first-line anti-TB drug, characterized as a highly effective, orally administered and non-toxic compound, as part of the combined therapy approach recommended by the WHO [23]. RIF has an effectiveness of about 95% in cases of TB caused by susceptible strains [47], with a daily oral administration of the standard 600 mg single dose [24,48] or of a dose corresponding to 10–20 mg RIF/kg body weight [49,50]. Alone or in combination with other drugs, it represents the main treatment for infections (produced by various microorganisms), such as TB (*Mycobacteria tuberculosis*), osteomyelitis (*Staphylococcus aureus*), meningococcal disease (*Neisseria meningitidis*), leprosy (*Mycobacterium leprae*), gonorrhea (*Neisseria gonorrhoeae*) [51], Legionnaire’s disease (*Legionella pneumophila)* [19,28] and even HIV [52,53]. Under regulated dosage, RIF is also recommended against methicillin-resistant *Staphylococcus aureus* [19], to treat cholestatic pruritus and infections associated with prosthetic joints [54], such as chemoprophylaxis of the postoperative endophthalmitis [55], or against Brucellosis and meningitis caused by *Streptococcus pneumoniae*, *Haemophilus influenzae* Type b and *Neisseria meningitides* [19]. It is generally used in combination with other antibiotics in order to prevent bacterial resistance of different microorganisms, to extend its antibacterial activity and, thus, to enhance its effectiveness towards various infections originating from both intracellular and extracellular organisms, as well as to improve acceptability and compliance [28,56]. For example, TB treatments include a two-month intensive therapy with different combinations of the first-line agents RIF, ISN, PYR and ETB, followed by another four months of RIF and ISN administration [57]. It was found that RIF could be applied for wound healing as well [58], and it was also explored to prevent the formation of biofilms [43]. There have been reports emphasizing that RIF has a neuroprotective function [59] and a therapeutic role in neurodegenerative disorders [60,61], including Alzheimer’s disease [62], as well as an ability to increase the accumulation of anticancer drugs in multidrug-resistant cancer cells [63], to cleavage or to bind to DNA [64], which may contribute to RIF anti-cancer activity. It must be mentioned that RIF is not used for the treatment of viral infections, such as colds or flu [28]. On the other hand, it was observed that RIF may significantly reduce the effects of many drugs by increasing their metabolic rate [19]. 

RIF is usually orally administered and it is rapidly absorbed in the gastrointestinal tract and distributed throughout the body [19,28]. Due to its liposoluble character, RIF easily penetrates into all tissues [57]. It is metabolized in the liver by deacetylation and is excreted in the bile together with its metabolites [65]. In healthy individuals, RIF half-life is around 2–3 h. After 6 h, the drug is completely eliminated from the body [28] through urine (about 30%) and mostly through feces (60–65%). Around 7% of the administered RIF is eliminated intact via urine [19].

Long-term or excess use of RIF may produce some adverse effects, the most important being hepatotoxicity, which causes liver damage leading, for example, to jaundice. Other side effects include fever [66], arthralgia [31], kidney failure [67], fatigue, dizziness, gastrointestinal disorders [68] generating nausea, vomiting, appetite loss, diarrhea, sore throat, headache, immunological [53] and allergic reactions [52]. Moreover, RIF causes orange coloration in urine, sweat and tears [28]. All of these negative aspects can lead to treatment failure and to the occurrence and even prevalence of (multi)-drug-resistant TB [48,69], especially in patients with HIV infection [56,70]. 

## 3. Rifamycins Delivery Systems

Rifamycins’ bacterial activity is proportional to its concentration at the target site. RIF has low solubility in water and, in biological fluids, this is an impediment to achieving optimal concentration and limits its bioavailability. Therefore, high doses (450–600 mg per day) of RIF are required for oral administration and prolonged therapy (at least 6 months) damages the liver and also disturbs other biological functions (e.g., renal, gastrointestinal, immunological, etc.). Studies have emphasized that both β-CyD and γ-CyD form inclusion complexes with RIF, thus enhancing drug solubility and the percentage of RIF released in in vitro studies, increasing its efficiency and reducing side effects by lowering the daily dose administrated in the treatment of bacterial infections [71]. Encapsulation of RIF in a carrier reduces the antibiotic adverse reactions and intensifies its therapeutic effect. For example, RIF was encapsulated in round biodegradable poly(betulin disuccinate-co-sebacic acid) microspheres with diameters in the range of 2.00–21.00 µm; 40.00–60.00% of RIF release from these polymeric microspheres was detected in the first 72 h and continued for about 1 month. Therefore, poly(betulin disuccinate-co-sebacic acid) microspheres could be used as RIF delivery systems [72]. Rifamycin S was encapsulated in niosomes prepared by a slightly modified thin film hydration method. The vesicles membrane consists of the surfactant Span^®^ 60 and cholesterol. Results of the minimum inhibitory concentration assay emphasized that the niosomes containing glycerol in the aqueous phase presented higher antibacterial activity [73]. MSNPs encapsulated with RIF were developed as nanocarriers to deliver the antibiotic into macrophages for the treatment of intracellular infections associated with SCV of *Staphylococcus aureus* [74]. A study regarding the influence of the surface chemistry and size of spherical MSNPs with controllable diameters < 100 nm and pore sizes > 5 nm revealed that 40 nm particles with high silanol groups density presented enhanced cellular uptake and sufficient drug loading, resulting in improved antibacterial efficacy of the encapsulated RIF against SCV of *Staphylococcus aureus* [75]. Porous biphasic calcium phosphate scaffolds coated with RIF-loaded biopolymers, poly(ε-caprolactone) or poly(ester urea), were developed as antibiotic delivery systems to avoid bacterial (*Staphylococcus aureus* and *Escherichia coli*) infections, with possible applications in bone repair [42]. A cefazolin-containing polycaprolactone 3D scaffold encapsulated in a RIF-loaded alginate hydrogel resulted in a dual-drug-releasing scaffold with a 5.00 mm diameter disk-shaped design. This system presented a synergistic effect due to the ability of the external antibiotic, RIF, to inhibit biofilm formation and, of the internal drug, cefazolin, to increase antibacterial activity against *Staphylococcus aureus* and could be used to amend the treatment of osteomyelitis [76]. RIF-containing polyelectrolyte nanoparticles acting as drug-delivery systems were synthesized in order to protect the drug against the alkaline medium of chronic wounds. The hydrophobic RIF was entrapped in CS modified with a hydrophobic amino acid (alanine or tryptophan). The amphiphilic cores were covered by means of the negatively charged polysaccharide, dextran sulfate, with the positively charged PEI bearing three types of amino groups, which are protonated at physiological and endosomal pH. The electrostatic interactions of the cross-linker dextran sulfate with the positive charged groups of both CS and PEI resulted in stable nanostructures. The study emphasized that these NPs and the antioxidant ascorbic acid have synergistic protective effects, improving the RIF release rate and decreasing its degradation rate in alkaline media (pH 8.40) [41]. 

Owing to their flexibility and soft nature, microfibers are used for drug delivery applications. In 2021, Sharma et al. [58] described the fabrication of RIF-loaded microfibers by the ionotropic gelation method, starting with the naturally occurring biopolymers sodium alginate and gelatin, to which xanthan gum and nanoclay were added to enhance the mechanical strength of the fibrous structure. These biocomposite microfibers could be used for controlled and prolonged RIF release and as wound dressing material. 

**Figure 2:**
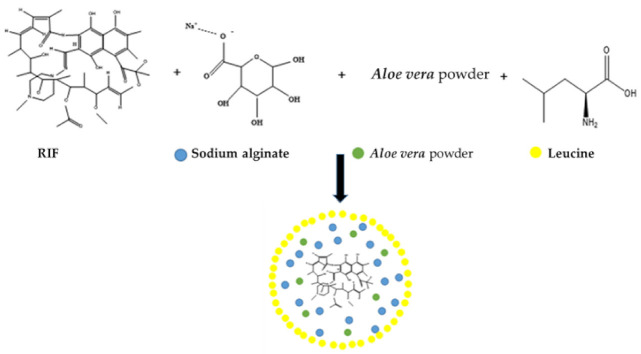
Schematic representation of drug, matrix former, spray drying excipient and spray-dried RIF-loaded microparticles (adapted from [38]).

Despite its disadvantages, the oral route of RIF administration remains dominant. Nevertheless, other methods of targeted delivery of the drug are being sought, which would increase the bioavailability of the antibiotic and reduce adverse reactions. Pulmonary administration of RIF is one of the alternatives to refine TB therapy due to the rapid onset of the drug action, high RIF concentration in the lungs, lower enzymatic activity, avoidance of hepatotoxicity and fewer systemic side effects. The development of particulate carriers for pulmonary drug delivery would enable controlled drug release and selective drug targeting to the desired location. Inhalable RIF-loaded, nearly spherical polymeric microparticles (Figure 2) with diameters of 2.00 to 4.00 µm and optimum aerodynamic characteristics concerning aerosolization and inhalation were prepared using the spray drying technique from sodium alginate, and aloe vera powder as a matrix former and L-leucine as a spray drying excipient. The drug content of the prepared microparticles varied from 0.24 to 0.39 mg/mg of powder, while the drug association efficiency (between 39.28 and 96.15%) was enhanced by increasing concentrations of both L-leucine and sodium alginate. Dissolution data obtained for these microparticles emphasized that, due to the hydrophobic nature of L-leucine, the in vitro release of RIF significantly decreased with increasing L-leucine concentration in comparison to the formulations with higher sodium alginate concentrations. The retarded dissolution of RIF from these microparticles offered enough time for their phagocytosis by alveolar macrophages, resulting in the elimination of *Mtb* present within the cells [38]. 

RIF-loaded polymeric micelles were developed for administration by inhalation of the antibiotic. The commercially available graft copolymer Soluplus^®^ (poly (vinyl caprolactam)-poly(vinyl acetate)-poly(ethylene glycol)) was used, and increased, by 14.30-fold, RIF aqueous solubility and generated nanosized micelles (~107 nm). This inhalable nanosystem was proven to multiply, up to 2.50-fold, in vitro RIF microbicidal efficacy against *Mtb* in comparison to a RIF solution, and it was shown that the antibiotic accumulated in rat lungs over 24 h [77]. More recently, applying the solvent diffusion method, RIF and curcumin were encapsulated in mannose surface-decorated Soluplus^®^ micelles. After freeze-drying, the drug-loaded polymeric nanomicelles (~108 nm) were stable after dilution in simulated interstitial lung fluid, being suitable for nebulization and drug delivery to the deep lung. The studies revealed that mannose enhanced the microbicidal activity of the inhaled micellar nanoformulation, while curcumin contributed with its anti-inflammatory and potential anti-microbial effects [48]. 

A mucoadhesive liquid crystal system composed of surfactant (ethoxylated and propoxylated cetyl alcohol), the RIF-containing oil phase consisting of oleylamine, soy phosphatidylcholine or a mixture of 1:1 (m/m) oleylamine / soy phosphatidylcholine and deionized water as the aqueous phase was developed for the nasal, sublingual, and cutaneous routes of RIF administration. The slow-drug release delivery nanostructured system was assessed in preclinical studies on *Mtb*-infected mice and could be used in TB therapy [47].

A RIF-loaded thermo-sensitive mucoadhesive in situ rectal gel was recently developed in order to improve the antibiotic’s bioavailability and to attenuate its hepatotoxicity. RIF solubility was increased by co-precipitation with PEG 6000 and was then incorporated into the mucoadhesive in situ rectal gel with the optimized composition of 0.10% RIF:PEG (1:1) co-precipitate, 25.00% Pluronics (copolymer of poly(oxyethylene)–poly(oxypropylene)–poly(oxyethylene); i.e., 10.00% of Pluronic F68, 15.00% of Pluronic F127 as a gel base and 1.20% of sodium alginate acting as mucoadhesive polymer [69].

Due to its efficacy in the treatment of bacterial infections, RIF was used in the synthesis of an antibacterial biocomposite polymeric system applied to a prophylactic covering of polypropylene mesh material employed for hernia repair. The coating, consisting of RIF-loaded poly(d,l-lactide-co-glycolide) biodegradable nanoparticles dispersed in CS, gradually released the drug for up to 11 days, being active against *Staphylococcus aureus* and *Staphylococcus epidermidis* for at least 14 days. The antibiotic-loaded biopolymer inhibited bacterial adhesion to the mesh material and presented good cell compatibility [78].

## 4. Rifamycins Detection and Monitoring

Considering RIF concentration-dependent toxicity and possible drug resistance, as well as the differences in the pharmacokinetics and its varied oral bioavailability [74] among different people, the TDM during treatment with RIF is very important. Therefore, reliable RIF quantification in biological samples allows for a trained physician to establish the optimal individualized doses to obtain the most effective treatment with the least adverse reactions [45]. The antibiotic can be detected in human blood serum or urine samples. The RIF therapeutic peak plasma concentration is attained at 2 h after oral administration. Due to RIF variable oral absorption, blood collection for TDM is recommended to be performed at 2 h and 6 h post dose ingestion [79].

The anti-TB treatment very often involves the administration of RIF together with other drugs, like ISN and PYR. Therefore, the selectivity and the sensitivity of analytical methods are compulsory features for simultaneous determination of the drugs in both pharmaceuticals and more complex matrices, like biological samples [18,56,68,69,80,81,82,83,84,85,86,87,88]. RIF bioavailability and, therefore, its therapeutic action can be influenced by its physical or chemical interactions with other molecules (peptides, DNA, drugs, nutrients, etc.). Various electrochemical [23,50,89,90] and spectrometric (optical or luminescence) [30,91,92,93,94] methods have been reported to be applied to investigate such interactions [43,95,96].

RIF is the active principle of several pharmaceuticals, most of them being in solid form because there is a lack of liquid or aqueous RIF pharmaceutical dosage forms due to low water solubility and high chemical instability [48]. However, the pharmaceutical industry has very strict regulations regarding the quality and stability of the products, with methods of analysis being decisive to monitor the composition of both final and intermediary products, throughout the whole fabrication process. In this respect, several simple methods were reported for RIF assessment in pharmaceutical dosage forms to ensure its concentration is within the effective range when administered [97].

RIF can enter into surface waters, groundwater and sediments as a result of leakage from manufacturing and usage processes, or through domestic sewage due to the extraction of intact or metabolized RIF after therapeutic administration. RIF has low water solubility and is environmentally stable, leading to persistent toxicity and distortion in the ecosystems, disruption of the endocrine functions of the aquatic organisms and humans and it causes antibiotic-resistant genes in pathogenic species in fish [37]. Considering the hazard posed by the persistent drugs in the environment, today there is an increasing trend in the development of various materials (green synthesized magnetic Fe_3_O_4_ nanoparticles [98], water/dimethylsulfoxide-transcutol/isopropyl alcohol/capmul MCM C8 nanoemulsion [99], graphene hydrogel/M (M:Cu, Co, Ni nanocomposite) as cathode [100], Co_3_O_4_ NPs supported on olive stone biochar [101], etc.) and processes (e.g., Fenton reaction, electrochemical process and their combination [102], electrochemical [103], etc.) for RIF removal from various water samples. 

### 4.1. Electrochemical (Bio)Sensors and Methods for Rifamycins Analysis

**Figure 3:**
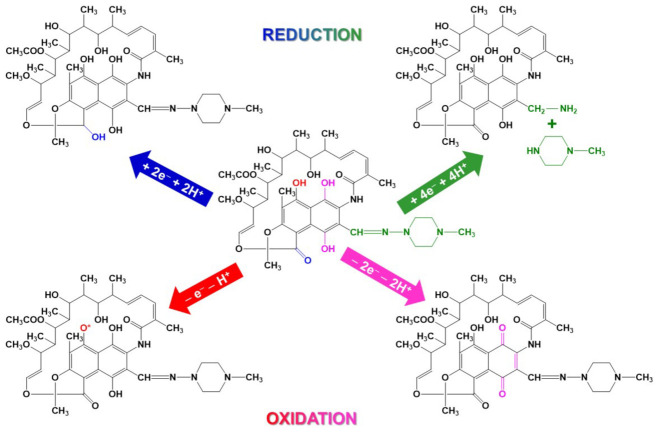
RIF electroactive sites and possible electrode reactions.

Rifamycins, in general, and RIF and RSV, in particular, have many functional groups, some of them being electroactive, so that the molecules can be either reduced or oxidized (Figure 3) at proper selected (bio)sensors. 

#### 4.1.1. Electroanalytical Methods Based on Rifamycins Electroreduction

RIF cathodic behavior was investigated and exploited in qualitative analysis using mercury electrodes such as DME [70], SMDE [34] and HMDE [25,56,83,104]. RSV quantification by adsorptive stripping voltammetry at HMDE was also reported [105].At mercury electrodes, RIF voltammograms presented two peaks assigned to the irreversible reduction of the azomethine (−C=N−) functional group from the side chain of the 3-[(4-methyl-1-piperazinyl) imino] methyl moiety, which is more easily reducible and generates the more intense signal observed at less negative potentials (~0.95 V at pH ~7.00), and of the carbonyl group (situated at ~1.20 V at pH ~7.00), respectively [34,83].

Few electroanalytical methods based on RIF reduction signals have been reported, and those that have been reported date from the early 2000s. Most of these methods presented relatively narrow linear ranges (from one to maximum three orders of magnitude) and LODs at submicromolar levels, which were lowered to the nanomolar level when adsorptive techniques were applied (Table 1). Today, due to mercury toxicity, this type of electrode is rarely used. However, to mimic the surface of mercury electrodes, a silver wire coated with a Hg film (HgFE/Ag) was used as a renewable working electrode with an adjustable surface area. The RIF peak current obtained by DPV in the same conditions was 2.5 times higher on HgFE/Ag than on HMDE, most probably due to the larger surface area of the HgFE/Ag [55].

#### 4.1.2. (Bio)Sensors and Electroanalytical Methods Applied for Rifamycins Electrooxidation Investigations

RIF and RSV (Figure 1) are hydroquinone derivatives (−OH groups in positions 1 and 4, Figure 1) and possess a third phenolic −OH moiety (position 8), which can be chemically or electrochemically oxidized [40] at various solid bare electrodes (e.g., GCE [85], CPE [80] and the disposable PGE [84]) or modified sensors obtained at various substrates, such as GCE [28,29,32,37,45,52,65,66,67,68,85,90,106,107,108,109,110,111,112,113,114,121], SPE [36,53,115], CPE [21,26,80,88,116,117], ITO [118], Ag [55], Au [50,86] and Pt [119,120]. Most often, the electrodes were employed as sensing devices in voltammetric [26,27,28,31,36,37,45,50,52,53,55,65,66,67,68,80,84,86,88,106,107,108,109,110,111,112,113,116,117,121], potentiometric [21], amperometric [114,119,120] or photoelectrochemical [108,115] analysis of RIF, while some of them have also been applied as detecting systems in high performance liquid chromatography [85].

Studies revealed that, depending on the nature of the sensor’s electroactive surface, RIF can present two [28,53] or three [26,36] oxidation signals (Figure 3). The signal at more anodic potential was irreversible, while the others were reversible [28,53], quasi-reversible [36,52,82,112,116] or even irreversible [67]. The (quasi)reversible couple involved an exchange of two electrons and two protons, and was related to the hydroquinone-quinone system of the 1,4-dihydroxynaphtalene structure [26, 27,28,31,36,52,53,65,82,110,116]. The signal situated at more anodic potential was assigned to the irreversible one electron/one proton oxidation of the piperazynil-imino moiety of RIF [26, 27, 28,53,110] or of the phenolic hydroxyl (position 8) [26,112,116]. RSV CV emphasized the quasi-reversible peak pair corresponding to the hydroquinone-quinone system [26,121] and an irreversible anodic signal at a high potential value attributed to the oxidation of the -OH group from position 8 (Figure 1) [26]. Rifamycin B presented only a single irreversible oxidation peak, as expected for the *O*-monosubstituted hydroquinone [121].

In order to improve its selectivity and sensitivity, the sensor’s electroactive surface was chemically or electrochemically modified with one or more materials (Table 1), leading to (1) an increased electroactive surface area [52,68] (e.g., the effective surface area of the DyNWs/CPE was approximately eight times larger than that of the bare CPE [116], that of the CoEnQ10/Fe_3_O_4_NPs/MWCNTs/GCE was about 3.3 fold as large as that of GCE [67] and bare GCE (0.068 cm^2^) < MoSe_2_/GCE (0.102 cm^2^) < rGO/β-CyD/GCE (0.138 cm^2^) < MoSe_2_/rGO/β-CyD/GCE (0.214 cm^2^) [37]) and (2) faster electron transfer by decreasing the charge transfer resistance [52,68,110,118] (e.g., GCE (368.94 Ω) > GMONRs/GCE (113.39 Ω) [31] or GCE (748.04 Ω) > MoSe_2_/GCE (354.58 Ω) > rGO/β-CyD/GCE (236.41 Ω) > MoSe_2_/rGO/β-CyD/GCE (105.02 Ω) [37]). 

The simplest way to change the electrode’s surface and thus its performance characteristics, is electroactivation, which can be performed either potentiostatically or potentiodynamically using different supporting electrolytes [122]. A SPBDDE was electrochemically pretreated by CV (five cycles, scan rate 0.100 V/s; potential range 0.000 to 2.000 V) in 0.10 mol/L NaOH, in order to generate hydroxyl radicals at its surface and to increase its reactivity towards RIF, so that a LOQ of 7.30 × 10^−13^ mol/L was achieved with AdSDPV [36].

For RIF electroanalysis, the literature contains few reports regarding sensors modified with a single material using different simple procedures. RIF oxidation was investigated at in situ electroplated Pb film on Au, boron-doped diamond and GC electrodes. Au could not be used as a substrate because RIF and Pb oxidation signals obtained by SWV were overlapped, so Pb/GCE was selected for further studies [106]. MIPs are often selected as electrode surface modifiers due to their ease of preparation, high stability and their unique tailored molecular recognition properties, which provide the modified sensor with high sensitivity and improved selectivity [1,123]. MWCNTs are widely used as electrode coating material, characterized by low cost, good conductivity, large surface area, extended potential window, low reactivity and mechanical strength [65,67,110]. Metal oxide nanomaterials have gained even more applicability in sensor development due to their large surface-to-volume ratio, excellent electrical and catalytic properties, their low cost and simple preparation methods [65,67]. A mixture of preformed polyphosphazene polymer and RSV [121] or nanomaterials, such as MWCNTS [28], GNPls [108], GMONRs [31] and BiVO_4_ microspheres [53], were drop-coated on GCE [28,31,108,121] or SPCE [53], and dried at air [28,108,121] or at a given temperature (e.g., 15 °C [31], 50 °C [53]). The preparation procedure of the MI polyphosphazene modified electrode comprised a supplementary step involving the extraction of the template (RSV) from the polymeric membrane by washing with ethanol [121].

HP-β-CyD/CPE [21] and DyNW/CPE [116] were obtained by simply hand mixing the modifier (HP-β-CyD and DyNW, respectively), carbon powder and paraffin oil, and filling the tip of a disposable polyethylene syringe [21] or a polytetrafluoroethylene tube [116] with the corresponding paste. The electrical contact with the carbon paste was realized by a stainless steel [21] or a copper [116] wire. The electrode surface was renewed by squeezing out a small part of the carbon paste from the holder, and the resulted new surface was polished on a special paper until it obtained a shiny aspect [21,116]. By immersing a CPE, for a given period of time, in a stirred solution containing the surfactant at a concentration lower than the critical micellar concentration resulted an in situ surfactant modified CPE. It is worth mentioning that, in this case, the electrode modification did not lead to better RIF or RSV detection sensitivity (Table 1); instead, it allowed the determination of RIF in the presence of RSV using an anionic surfactant (SDS)-modified CPE and of the RSV in the presence of RIF when the CPE was modified with the cationic surfactant CTAC. This can be explained by the electrostatic interaction between the antibiotics and the modified CPE, depending on the charge of the analyte at a given pH, e.g., at pH 2.00 RSV is neutral, while RIF is protonated at the piperazine moiety and, therefore, it is attracted by the negatively charged groups of SDS [26]. 

The sensor’s performance characteristics are most often much better if their surface is modified with a combination of various materials (e.g., carbon-based nanomaterials, metal oxides, polymers, different organic species, etc.) due to their synergistic effects. 

**Figure 4:**
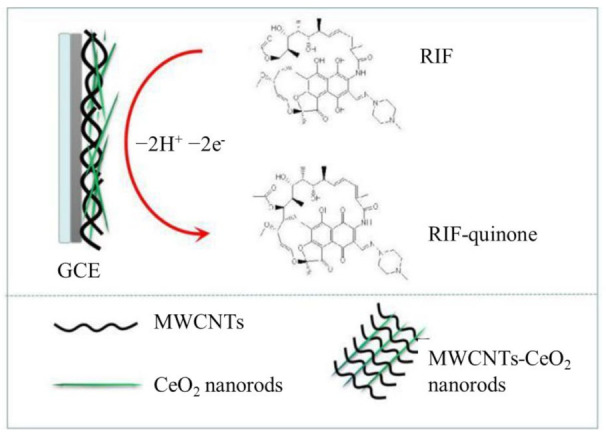
The scheme of the “nanonetwork” modified electrode and RIF electrooxidation at its surface [65].

Nanocomposites of MWCNTs with CeO_2_ NRs [65] (Figure 4), Fe_3_O_4_NPs [67], lecithin stabilized SPIONs [111], Mo_2_C [109] and CoTHPP [110] were used in the development of RIF sensors. To further increase the sensor electrochemical performances for RIF detection, coenzyme Q10 was immobilized by drop-casting at the Fe_3_O_4_NPs/MWCNTs/GCE. For the series of stepwise modified sensors, RIF anodic peak currents recorded in the same conditions were GCE (28 µA) < MWCNTs/GCE (75 µA) < Fe_3_O_4_NPs/MWCNTs/GCE (150 µA) < CoEnQ10/Fe_3_O_4_NPs/MWCNTs/GCE (220 µA) [67]. 

Graphene and its derivatives (GO and rGO) have also been frequently used as electrode modifiers due to their large surface area, high hydrophilicity and good mechanical strength [19]. For example, the RIF sensors MoSe_2_/rGO/β-CyD/GCE [37], CuO@rGO/GCE [52], TiO_2_/rGO/GCE [66] and NiTAPc-GO/ITO [118] were simply obtained by drop-casting a 2:1 ratio of MoSe_2_:rGO/β-CyD solution, the homogeneous CuO@rGO composite solution, an ethanolic solution of TiO_2_/rGO and Nafion and a NiTAPc-GO suspension in DMF, respectively, onto the corresponding substrate surface and drying in an oven [37] or at room temperature [52,66,118]. However, covering the substrate surface with different types of modifiers may involve more steps. Ni(OH)_2_NPs-rGONSs/GCE was prepared by applying a layer-by-layer procedure consisting of drop-coating a GO film onto the GCE, followed by potentiostatic reduction (200 s at −1.200 V in ABS pH 5.00) to rGONSs and the subsequent in situ synthesis of Ni(OH)_2_ NPs on the rGONSs/GCE through electrochemical reduction (100 s at −1.000 V) of Ni(NO_3_)_2_ in ABS pH 5.00. Ten additional CV scans were performed in 0.10 mol/L NaOH solution in order to assure the electro-dissolution and passivation of the Ni(OH)_2_ film, resulting in the desired modified sensor, which was daily prepared. The GCE modification led to the reduction of the peak potentials and the enhancement of the currents for the RIF two anodic signals (by a factor of 20 and 27, respectively), which were due to the increased surface area of the Ni(OH)_2_ NPs, the high density edge plane-like defects and oxygen-containing groups of the rGONSs, providing more active sites that favor the RIF adsorption and the electron transfer between the RIF and the electrode [27].

Simple or MI polymeric films are among the preferred modifiers in order to attain better electrochemical characteristics of a sensor, one of the most used being PPy. In their work, Alonso Lomillo et al. [119,120] started the Pt surface modification by covering it with a thin PPy layer electrogenerated by CV. In the subsequent step, the HRP-PPY [119] and β-CyD-PPy [120] films, respectively, were deposited on the PPy/Pt electrode by CV from a Py solution containing the corresponding modifier. LiClO_4_ was always used as the supporting electrolyte. A layer of PPy containing RIF and ISN as templates was electrodeposited by CV on a GCE previously modified by drop-casting with Cu-MOF/MC mixture and dried under IR radiation. After removing the template molecules by extraction in a methanol/water mixture, the dual MI-modified electrode, MIPPy/Cu-MOF/MC/GCE, which presented for both analytes linear ranges of three orders of magnitude and LODs lower than 1 nmol/L, was used for the simultaneous quantitative determination of RIF and ISN from various matrices [68]. 

According to the literature, the most sensitive electrochemical determination of RIF was reported to be by AdSSWV using a GCE modified by drop casting with CoFe_2_O_4_@CdSe core-shell NPs capped with the biodegradable polymer PVP. The wide linear range of nine orders of magnitude and the very low LOD (see Table 1) and LOQ (1.52 × 10^−16^ mol/L) values were attributed to the high catalytic effect and the mesoporous structure of the CoFe_2_O_4_@CdSe NPs, which provided many active sites for RIF accumulation. On the other hand, RIF concentration at the electrode surface was increased by the strong analyte adsorption via the hydrogen bonds formed between the hydroxyl and amino groups of RIF and the PVP carbonyl groups [112]. 

#### 4.1.3. (Bio)Sensors and Electroanalytical Methods Applied for Rifamycins Indirect Determination

An interesting procedure for NiHCF/GCE preparation involved more steps, namely: the electrode surface was covered with Ni NPs by drop-coating and the obtained electrode was cycled (3–300 times) in the potential range 0.000 to 1.200 V in a 1.00 × 10^−3^ mol/L K_3_[Fe(CN)_6_] in 0.10 mol/L KCl solution. RIF determination at this electrode was due to the antibiotic interaction with the Ni species from the electrode surface, resulting in a decrease in the NiHCF reduction signal, this variation being proportional to RIF concentration [107]. 

An enzymatic biosensor was prepared by potentiodynamic electropolymerization of pyrrole in the presence of HRP using a Pt electrode. The function of the HRP/PPy/Pt is based on a “ping-pong” or double-displacement mechanism involving H_2_O_2_ and RIF as substrates. The enzymatic reaction, through which RIF is oxidized to the RIF-quinone form, is monitored by measuring the RIF-quinone reduction current, which is proportional to the RIF concentration in solution if the concentration of H_2_O_2_ is high enough so that it is not a limiting factor [119]. 

A nanobiosensor was obtained by the potentiostatic electrodeposition (at 0.700 V) of the EG-CYP2EI onto the Au electrode modified with the PVP-AgNPs/PANSA nanocomposite, due to the electrostatic interactions between the enzymatic system and the oxidized nanocomposite. Considering RIF multiple interactions with the CYP2EI ferri-heme, this biosensor was applied to investigate RIF biotransformation into its carboxylic form, via formylrifampicin. CVs recorded at EG-CYP2E1/PVP-AgNPs/PANSA/Au presented an irreversible cathodic signal at −0.300 V, for which intensity linearly varied with RIF concentration. Due to the fact that RIF peak serum levels are comprised within the linear range of this nanobiosensor, it could be used to detect RIF in serum samples using DPV or amperometry [50]. Another enzymatic biosensor, constructed in a two-step drop-coating process via physical adsorption of CuPPI onto an Au electrode and the subsequent electrostatic attachment of CYP3A4, was developed for detection in aerobic conditions of the four anti-TB drugs PYR, ETB, ISN and RIF. The biosensor reduction peak at −0.080 V, attributed to monooxygenation reaction occurring within the enzyme, linearly increased with the added drug concentration due to the interaction between the antibiotic and the Fe(III) active site of the enzyme. The biosensor had the highest sensitivity for PYR (3000 A × L/mol) and the lowest for RIF (890 A × L/mol), with the corresponding LOQs of 6.80 × 10^−11^ mol/L PYR and 3.25 × 10^−10^ mol/L RIF [86].

RIF electrochemical behavior has enabled its use as a mediator in H_2_O_2_ reduction. When it was immobilized onto a CoFe_2_O_4_@CdSeQDs/GCE, the CVs recorded in blank solution at the resulted CoFe_2_O_4_@CdSeQDs/RIF/GCE showed the RIF characteristic pair of redox peaks. In the presence of H_2_O_2_, the RIF cathodic peak was augmented, while the anodic one decreased, indicating that H_2_O_2_ diffused towards the electrode and oxidized the reduced form of RIF. The linear dependence between the decrease of RIF cathodic peak current and H_2_O_2_ concentration enabled the amperometric detection of H_2_O_2_ in the concentration range 7.00 × 10^−6^–1.43 × 10^−3^ mol/L [124]. 

### 4.2. Spectrometric Methods for Rifamycins Analysis 

#### 4.2.1. UV-Vis Spectrometric Methods Applied to Rifamycins Analysis

UV-Vis spectrometric methods are routinely used due to their simplicity and the need for less expensive instrumentation. However, their sensitivity and selectivity are not as performant as those of other instrumental methods of analysis.

RIF was determined in the presence of other drugs from combined dosage forms, without any pretreatment step, by UV spectrometric measurements at the wavelengths corresponding to their maximum absorbance, based on the fact that the mixture absorbance is additive at any wavelength [125,126]. On the other hand, some reports described the “zero-crossing” method applied to the first order derivative spectra. In this case, RIF was determined at the zero-crossing point of the other(s) coexisting analyte(s), where it presented a linear ΔA/Δλ = f (C) dependence [126,127]. The Q-AR method was also reported to be applied for the quantification of RIF in the presence of another compound (e.g., piperine [128] and CC-I [129]). This method employs the ratio of absorbancies at the wavelengths’ corresponding to the iso-absorptive point and to the maximum absorption of one of the analytes. Youseff and Mahler [130] created a short mathematical explanation of the basic principles of the DRSZ, DDRD and HDDR methods, and optimized each of them for RIF, PYR and ISN quantification in ternary mixtures without prior separation. Chemometric methods like PLS [131,132], PCR and CLS multivariate calibrations were also reported for RIF determination from mixtures with piperine [133], RSV [131] and ISN [132,134]. 

An older study worth mentioning reported that when RIF and ISN are present in fixed-dose combinations, they interact and form isonicotinylhydrazone, which has a similar visible spectrum with RIF and its absorptivity is one third of that of RIF, so that colorimetric determination can lead to an overestimation of RIF content with 33% [135].

A RIF assessment from its mixture with ISN and PYR was based on the fact that RIF is soluble in ethyl acetate and not soluble in water, while the solubility of the other two analytes is reversed. Therefore, after extraction in ethyl acetate, RIF was determined by simple absorbance measurements at 334 nm [136]. RIF was quantified from wastewater samples using the standard addition method and the absorbancies measured at 338 nm, after cloud point extraction in the presence of the surfactant Triton X-100 [137]. However, considering the RIF absorption maximum at 474 nm, it was directly determined in the presence of ISN without any interference [138]. 

There are several methods based on RIF reactions with one or more reagents in order to obtain colored products for which the absorbance is measured, either in the reaction medium, e.g., charge transfer complexes [139,140], or after extraction into a proper solvent [139]. 

Indirect spectrometric methods were based on RIF reaction with NBS through quantification of the unreacted NBS after treatment with KI and monitoring the released I_2_ by absorbance measurements at 572 nm [138] or on the Fe(III) reduction by RIF to Fe(II), which was subsequently complexed with ferricyanide to form Prussian blue, for which absorbance was measured at 760 nm [140]. RIF and ISN simultaneous quantification from a mixture were performed by treating the sample with Cu(II) and neocuproine, and applying PLS regression to the spectrometric data recorded at 455 nm. The principle of this determination relies on the ISN ability to reduce Cu(II) in the presence of neocuproine to Cu(I) in the Cu(I)-neocuproine complex, which presented an absorption maximum at 455 nm. In these conditions, the RIF absorption maximum appears at 449 nm and, due to the peaks overlapping, a chemometric approach was necessary to determine the two drugs [132]. Additional details regarding these examples are presented in Table 2.

UV-spectrophotometry also proved to be a reliable method for the investigation of forced RIF degradation under acidic, alkaline, thermal, peroxide and photolytic stress conditions [141], and it was also quite often employed to investigate RIF in vitro release from newly developed drug carriers. UV-Vis spectra recorded at different time intervals for RIF in physiological (pH 7.40) and alkaline (pH 8.50) media in the absence and in the presence of ascorbic acid were exploited to investigate drug degradation and the protective role of the antioxidant. The study revealed that, at both pHs, RIF presented two absorption maxima, at 334 nm and 475 nm, which were almost unchanged during the first 6 h and decreased afterwards due to drug degradation. In the presence of ascorbic acid, the peaks drastically increased after 24 h, indicating the formation of a byproduct affecting the absorbances at the considered wavelengths. The changes in the absorbances at these two wavelengths recorded for RIF-loaded polyelectrolyte NPs were helpful to establish that the drug was gradually released for 24 h, and the release rate remained constant for 48 h [41]. UV-Vis spectrometry based on the RIF absorption maximum at about 330–340 nm was employed to monitor the in vitro antibiotic release from drug-loaded polymeric microparticles [38] or biopolymers coated on biphasic calcium phosphate scaffolds, while the RIF content of the corresponding polymers was assessed after the complete antibiotic dissolution, using the calibration curve method plotted in the concentration range 1.00–100.00 µg/mL in PBS and in DMSO, respectively [42]. The efficiency of RIF entrapment in microfibers [58] and its in vitro release from a dual-drug-based scaffold [76] were evaluated using similar methodologies. In vitro RIF release studies from biodegradable polymer microspheres during defined periods of time (1 h to 1 month) were performed by absorbance measurements in PBS at 470 nm applying the calibration curve method. The identical absorption spectra recorded for fresh solutions of RIF and for RIF released after different times suggested that there was no drug degradation during the encapsulation and release periods [72]. Absorption measurements at the same wavelength were reported to be used for the evaluation of the RIF-PEG 600 co-precipitate solubility in comparison to that of pure RIF and of the physical mixture [69]. The loading capacity and encapsulation efficiency of RIF in MSNPs [74,75], as well as its in vitro release from these nanocarriers, were assessed from UV-Vis measurements at 254 nm in methanol [74].

In methanol-water medium, RIF formed with UO_2_^2+^ a 1:1 yellow complex, with an absorption maximum at 375 nm. The linear dependence between the complex absorbance at 375 nm and uranyl ion concentration enabled the quantitative determination of UO_2_^2+^ from soil in the concentration range 1.35–20.25 µg/mL with LOD and LOQ of 0.20 and 0.61 µg/mL, respectively [144].

#### 4.2.2. IR Spectrometric Methods Applied to Rifamycins Analysis

IR spectrometric methods are not commonly used for quantitative determinations. Their main application is related to the fact that IR spectra give useful information regarding the functional groups existing in the molecules, the purity of a material [38], the degradation of a compound or its interaction with other chemical species (e.g., mannose with gelatin [48], RIF incorporated into alginate-gelatin microfibres [58] or RIF with PEG 600 [69] and with CyD [71]) being monitored based on the presence or disappearance of characteristic peaks (e.g., for RIF in the ATR-FTIR spectrum at 3477 cm^−1^ (−OH), 1728 cm^−1^ (furanone), 1644 cm^−1^ (amide near C−O), 1567 cm^−1^ (C=C), 1490 cm^−1^ (amide close to C−C) [38]). Comparison of the ATR-FTIR spectra recorded for RIF, the used raw materials (aloe vera, sodium alginate, L-leucine) and the resulted RIF-loaded microparticles confirmed the presence and the integrity of the raw materials during the synthesis process [38]. Similarly, the chemical composition, the functional groups and the interactions in the polyelectrolyte NPs were determined by analyzing the FTIR spectra of the pure individual components (CS, alanine, tryptophan, dextran sulfate and PEI), mixtures of them and of the NPs [41]. Even more, high throughput FTIR in combination with various machine learning algorithms proved to be suitable for the discrimination of the mechanism of action of 14 antibiotics (including RIF) at pathway, class and individual antibiotic levels. Despite the fact that this approach was successful for most of the investigated drugs, the prediction of the RIF mechanism of action was not effective. An explanation was that RIF is a RNA synthesis inhibitor and the used model did not know the metabolic fingerprint of RNA synthesis inhibition [94].

NIR spectroscopy coupled with PLS regression was applied to the simultaneous determination of RIF, ISN and PYR in tablets. The spectral ranges used for the determination were 1981–2195 nm for RIF, 1540–1717 nm and 2086–2197 nm for ISN and 1460–1537 nm, 1956–2022 nm and 2268–2393 nm for PYR [145]. NIR (spectral range 1100–2500 nm) and multivariate calibration by PLS analysis were employed to assess the percentage dissolution of RIF, ISN, PYR and ETB from pharmaceutical preparations [146]. The PLS approach was also used in the Raman spectroscopic simultaneous quantification of these four drugs in fixed-dose combination products [147].

### 4.3. Rifamycins Analysis Based on Luminescence Sensors and Methods

#### 4.3.1. Fluorescence-Based Sensors and Methods Applied to Rifamycins Analysis

**Figure 5:**
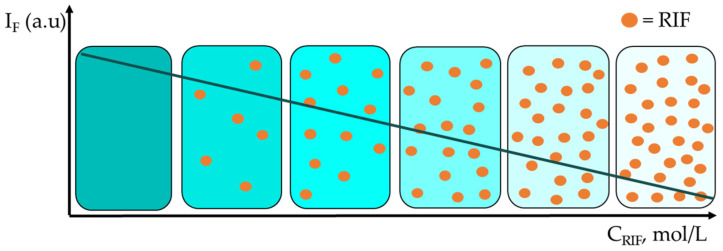
Schematic representation of RIF determination based on its fluorescence quenching ability.

Due to its inherent sensitivity, fluorimetry is often the choice for determinations at trace level. It can be directly applied either in the case of fluorescent analytes, or the species of interest can be determined based on their ability to quench the fluorescence of a fluorophore, as is the case for rifamycins. The fluorescence of various nanomaterials linearly decreased with the addition of increasing RIF concentrations (Figure 5) due to either a dynamic, as was the case for QDs [148,149], or static types of quenching, as in the situation of RIF interaction with HAS [30,92,150], with Cu NCs capped with PEI [151] or with FA [152]. When the UV-Vis spectrum of RIF, acting as a quencher, overlapped with the emission and/or excitation spectrum of the fluorophore, the inner filter effect (e.g., RIF interaction with N-P-CNDs [97], FA-Cu NCs [152], GSH-Cu NCs [153], UCCS NaYF_4_:Yb, Tm@SiO_2_ [154]) or the fluorescence resonance energy transfer (when CDs were the fluorophore [155]) might represent the main quenching mechanism. In the case of interactions of different rifamycins with serum albumins, the efficiency of fluorescence quenching is enhanced by the bulkiness of the R_2_ substituent, and therefore, it decreases in the order RFPT > RIF > RFD > RSV [30,93]. The performance characteristics of the fluorescence sensors reported in the literature for rifamycins determination are presented in Table 3.

Most fluorescence-based methods were employed to quantify RIF in capsules. Nevertheless, there are two interesting applications as a portable point-of-care medical diagnostic platform for RIF detection in urine. The two systems were obtained by immobilization of the BSA-Au NCs on wax-printed chromatography paper [54] and the UCCS NaYF_4_:Yb, Tm@SiO_2_ nanocomposite powder on filter paper using double sided sticky tape [154], respectively. Moreover, after its use, the latter system can be recycled and reused five times by simply ultrasonic washing with water. The RIF fluorescence quenching ability was also exploited in biodegradable poly(ethylene oxide) monomethyl ether-poly(ε-caprolactone) block copolymer NPs loaded with RIF containing a FRET sensor. The block copolymer was labeled with coumarin-based fluorescent dyes (C120 and DACCA). The principle of the RIF-based FRET sensor relies on the fact that FRET occurs if the energy donor (the coumarin-based fluorophores) and the energy acceptor (the quencher RIF) are sufficiently close (up to 10 nm) and if there exists significant overlap between the emission spectrum of the fluorophore (λ_em,C120_ = 440 nm; λ_em,DACCA_ = 482 nm) and the absorption spectrum of the acceptor (λ_abs,RIF_ = 477 nm). The sensor was applied to monitor real-time drug release, both in vitro and in living macrophages, and, in situ, the enzymatic degradation of the NPs in macrophages [156].

#### 4.3.2. Chemiluminescence-Based Sensors and Methods Applied to Rifamycins Analysis

By reason of its highly sensitive detection, wide dynamic ranges and simple instrumentation required, CL is often a good technique to be applied for monitoring chemical reactions or disease diagnosis. It relies on producing light following a chemical reaction which can be enzymatically catalyzed.

Unfortunately, there are few literature reports related to CL determination of RIF. The catalytic decomposition of peroxomonosulfate in the presence of Co^2+^ ions produces electromagnetic radiation, the intensity of which can be enhanced by the presence of RIF. The linear dependence between the CL intensity of this reaction and RIF concentration was used to develop a FI-CL method. The possible mechanism of CL amplification by RIF involved the oxidation of the RIF hydroxyl groups by the main reactive species produced by the HSO_5_^−^ /Co^2+^ system, resulting in an excited oxy-RIF, which returns to the ground state by emission of radiation [49]. In alkaline medium, RIF is degraded to RIF-quinone, which participates in a CL reaction with NBS acting as the oxidant; with the CL intensity depending on the degradation rate. The CL of ISN did not change over time. The different kinetic spectra of the two analytes combined with an artificial neural network calibration enabled simultaneous determination of the two drugs [81]. A CL sensor for RIF analysis was based on the drug’s ability to decrease the B,N-doped CDs enhanced CL intensity of the SO_3_^2−^ − Ce^4+^ system. This was due to the fact that RIF is easily oxidized and it competes with SO_3_^2-^ and B,N-doped CDs for the Ce^4+^, so that a lower amount of excited B,N-doped CDs was produced and the CL intensity decreased [44]. 

On the other hand, ECL couples the advantages of CL and electrochemical analysis, presenting high selectivity, simple in situ preparation and better reagent stability. In this technique, the species are generated at the surface of an electrode and then suffer electron-transfer reactions in order to form excited states that are finished by emitting light when they go back to the ground state. For example, in KH_2_PO_4_–Na_2_B_4_O_7_ buffer pH 6.60 - dodecyltrimethylammonium chloride solution, RIF was electrooxidated to a RIF-semiquinone radical intermediate, which was further oxidized by K_2_S_2_O_8_ to an excited state RIF-quinone and returned to the ground state with emission of radiation (460 – 575 nm), thus generating a detectable ECL signal [40].

It was also reported that, in borax buffer, when a potential of 1.200 V was applied to the working electrode, RIF had an amplification effect on luminol ECL signal. Dissolved oxygen and the RIF electrooxidation product were the key species responsible for the enhancement of the luminol ECL signal, which had a maximum intensity at 425 nm and was emitted by the excited state of 3-aminophthalate [157]. Other details regarding these examples are presented in Table 4.

## 5. Sensors and Analytical Methods for the Investigation of Rifamycins Interaction with Biological Important Molecules

Electrochemical and spectrometric investigations of interactions between a drug and biologically important molecules are of special interest because they help us to understand the drug’s behavior in physiological fluids and to elucidate their pharmacological action mechanism. 

CV and SWV studies at HMDE emphasized that the intensity of the anodic and cathodic peaks of rutin decreased and the peak potentials changed in the presence of RIF due to the formation, by electrostatic attraction, of an electroinactive 1:1 supramolecular RIF-rutin complex. These data were supported by results obtained by UV-Vis and FTIR measurements [95]. The same voltammetric techniques explain the formation of 1:1 adducts between RIF and cysteine and gluthatione, respectively, to propose a mechanism for these processes and to calculate the binding constants. The adducts formation was confirmed by FTIR analysis based on the disappearance of the free thiol group stretching band [96]. 

The interaction of RIF with ds- and thermally denaturated ss-calf thymus DNA was investigated both in solution and at DNA-modified CPE using transfer AdSDPV in ABS pH 5.00 and PBS pH 7.40. The investigation was based on monitoring the intensity of the guanine and adenine anodic peaks in the presence of different RIF concentrations after various interaction times when drug intercalation took place [89]. The interaction of RIF and NRIF with ds-DNA was studied by DPV at SPE modified with PB_290_-*b*-PDMAEMA_240_ diblock copolymer and MWCNTs. The binding constants of RIF-ds-DNA (1.48 × 10^4^ L/mol/8.56 × 10^4^ L/mol) and NRIF-ds-DNA (2.51 × 10^4^ L/mol/1.78 × 10^3^ L/mol) complexes assessed, based on adenine / guanine oxidation signals, confirmed the intercalation mode of RIF interaction with ds-DNA and suggested a mixed type of interaction (intercalation and electrostatic) in the case of NRIF-ds-DNA formation due to the lower binding constant obtained based on guanine anodic signal [23]. 

ATR-FTIR studies were used to investigate the mechanism of interaction between RIF and the membrane of neutral (dipalmitoylphosphatidylcholine) and anionic (dipalmitoylphosphatidylcholine:cardiolipin) liposomes. It was found that the main binding sites were phosphate and carbonyl groups of the lipids [158].

There are several studies regarding investigations by fluorescence spectroscopy of RIF interactions with both BSA and HSA. They are based on the RIF ability to quench the natural fluorescence of serum albumin given by the protein tryptophan residues (λ_ex_ = 285 nm and λ_em_ = 350 nm), the mechanism being a static one due to the chemical component (hydrogen bonding, hydrophobic effects, van der Waals’ interactions) between the molecules [30]. The association constant between RIF and BSA [91] and HSA [150] were found to be 1.83 × 10^5^ L/mol and 5.15 × 10^4^ L/mol, respectively. The number of binding sites of RIF with BSA [91,92] and of RSV with BSA and HSA is one; i.e., one rifamycin molecule binds to a tryptophan residue in the polypeptide chain. The binding is realized by hydrogen bonds between the rifamycins’ phenolic group and the tertiary amino group of the indole ring in tryptophan and the hydroxyl group in the rifamycins’ alkyl chain with the carboxyl group of tryptophan [30].

## 6. Conclusions

The literature data presented in this review emphasize that there is a relatively high interest in the development of sensors and instrumental methods for analysis of rifamycins, especially RIF. Most of these methods (almost one third) made use of electrochemical detection. This can be explained not only by the fact that electrochemical methods have the advantage of being relatively simple, rapid and cheap, but also through the multitude of materials and procedures that allow for the modification of the electrode surface in order to achieve more sensitive and selective determinations. Comparing the RIF therapeutic peak plasma concentration of 8–24 µg/mL (9.72 × 10^−6^–2.92 × 10^−5^ mol/L RIF) [159] with the linear ranges of the electrochemical methods reported in the last 20 years for RIF quantification (Table 1), it can be observed that, with one exception [90], all these methods could be applied to RIF blood level monitoring, most of them [19,25,26,27,28,36,45,50,52,56,65,66,67,83,84,86,88,104,106,111,112,114,115,116,117,120,121] needing a previous dilution step to bring RIF concentration into the linear range of the method. UV-Vis spectrometric methods present narrower linear ranges and are less sensitive; therefore, according to the data summarized in Table 2, most RIF determinations based on absorbance measurements may be applied as reported for the assessment of RIF blood concentration. Only a few of them required sample dilution [127,129,133], while the methods using a reagent p-chloranil or Fe(III) [139] are not applicable for such types of analysis. Regarding luminescence-based methods (Table 3 and Table 4), only one cannot be applied to RIF determination in blood samples [151]. Most of the others can be used as described, with a few exceptions [44,49,153,157], which require sample dilution. 

The LODs of about 70% of the electrochemical methods reported for RIF determination are comprised in the range 1.00 ×10^−9^–1.00 × 10^−7^ mol/L, with few of them achieving LODs lower than 2.2 × 10^−7^ mol/L [21,50,96]. While UV-Vis spectrometric methods are less sensitive, most luminescence-based methods presented LODs below 8.50 × 10^−8^ mol/L RIF. 

From the point of view of linear range and sensitivity, the majority of the electrochemical and spectrometric methods are suitable for the determination of rifamycins from blood samples and pharmaceutical preparations. 

This paper presents a brief overview of some recently published rifamycins delivery methods, with the aim to emphasize the importance of these antibiotics and the continuous concern for improving their efficacy. On the other hand, UV-Vis and IR spectrometric methods constitute valuable tools employed during the development of these drug carrier systems to investigate RIF stability and its release from them. 

The review also summarizes the instrumental methods reported for the investigation of RIF interaction with various biologically significant molecules.

Due to the fact that this is an up-to-date, comprehensive critical review on different types of (bio)sensors developed for rifamycins analysis, it is intended to give an overview on the topic for interested researchers and to inspire them toward further developments in the field. 

Improvements may be brought in: (i) the selectivity of rifamycins analysis methods (for example, by using MIP-modified sensors) and (ii) the sensors surface regeneration or even avoiding the fouling or the destroying of the sensitive part of the sensor. 

It would be of great practical interest to prepare easy to use, disposable sensors (e.g., paper-based devices, screen-printed or pencil graphite electrodes) to be applied in: (i) portable point-of-care testing systems involving non-invasive analysis (e.g., for urine or saliva samples) or (ii) flow systems for on-site environmental samples analysis (e.g., water).

## Figures and Tables

**Table 1:** The performance characteristics of electrochemical sensors reported in the literature for rifamycins determination.

Electrode	Technique	LR (mol/L)	LOD (mol/L)	Sample	Ref.
Rifampicin (RIF)					
DME	DPP	1.00 × 10^−7^–1.00 × 10^−4^	-	Spiked human serum	[70]
SMDE	DPP	1.00 × 10^−7^–1.00 × 10^−5^	1.00 × 10^−8^	Pharmaceuticals	[34]
HMDE	SWP_OFP_ SWP_1D_	6.00 × 10^−7^–1.21 × 10^−5^ 6.00 × 10^−7^–1.21 × 10^−5^	2.42 × 10^−7^ 2.59 × 10^−7^	Pharmaceuticals; urine	[56]
HMDE	DPV	4.87× 10^−7^–2.43 × 10^−6^	2.31 × 10^−7^	Pharmaceuticals	[83]
HMDE	AdSDPV AdSSWV	3.32 × 10^−8^–3.85 × 10^−7^	6.13 × 10^−9^ 9.83 × 10^−9^	Pharmaceuticals; urine	[104]
HMDE	AdSDPV Cu(II) complexation	1.99 × 10^−6^–2.78 × 10^−6^	1.70 × 10^−7^	Pharmaceuticals; urine	[25]
HgFE/Ag	DPV	4.87 × 10^−7^–3.00 × 10^−4^	1.46 × 10^−7^	Pharmaceuticals	[55]
GCE	HPLC/ECD	1.00 × 10^−5^–1.00 × 10^−1^	5.00 × 10^−10^	Pharmaceuticals; urine	[85]
Pb/GCE	AdSSWV	2.50 × 10^−10^–1.00 × 10^−8^	9.00 × 10^−11^	Pharmaceuticals	[106]
NiHCF/GCE	CV	5.00 × 10^−6^–5.00 × 10^−4^	2.60 × 10^−6^	Simulated human urine	[107]
MWCNTs/GCE	CV	5.00 × 10^−4^–4.00 × 10^−3^	5.00 × 10^−4^	Blood component	[90]
MWCNTs/GCE	DPV SWV	4.00 × 10^−8^–1.00 × 10^−5^	7.51 × 10^−9^ 1.13 × 10^−8^	Pharmaceuticals	[28]
GNPls/GCE	CV; DPV	1.00 × 10^−9^–1.00 × 10^−4^	5.00 × 10^−10^	-	[108]
GMONRs/GCE	DPV	1.50 × 10^−7^–1.36 × 10^−4^	7.10 × 10^−8^	Pharmaceuticals; serum; urine	[31]
MWCNTs-CeO_2_ NRs/GCE	DPV	1.00 × 10^−13^–1.00 × 10^−6^	3.40 × 10^−14^	Human serum	[65]
MWCNTs–Mo_2_C/GCE	-	5.00 × 10^−7^–7.40 × 10^−5^	4.50 × 10^−8^	Pharmaceuticals; human serum	[109]
MWCNTs-CoTHPP/GCE	CV	1.00 × 10^−8^–5.00 × 10^−3^	8.00 × 10^−9^	Pharmaceuticals	[110]
MWCNT-SPION/GCE	CV	1.00 × 10^−6^–6.00 × 10^−6^	1.18 × 10^−7^	Spiked human urine	[111]
CoEnQ10/Fe_3_O_4_NPs/MWCNTs/GCE	DPV	2.00 × 10^−6^–2.00 × 10^−5^	Peak I 3.20 × 10^−8^ Peak II 4.13 × 10^−7^	Pharmaceuticals	[67]
CuO@rGO/GCE	CV_anodic_ CV_cathodic_ DPV	5.00 × 10^−8^–3.50 × 10^−5^ 5.00 × 10^−8^–2.90 × 10^−5^ 5.00 × 10^−8^–2.55 × 10^−5^	6.00 × 10^−9^ 8.00 × 10^−9^ 6.00 × 10^−9^	Pharmaceuticals	[52]
TiO_2_/rGO/GCE	DPV	1.00 × 10^−11^–1.00 × 10^−10^	3.00 × 10^−11^	Pharmaceuticals	[66]
Ni(OH)_2_NPs-rGONSs/GCE	LSV_peak I_ LSV_peak I_	6.00 × 10^−6^–1.00 × 10^−5^ 4.00 × 10^−8^–1.00 × 10^−5^	4.16 × 10^−9^ 2.34 × 10^−9^	Pharmaceuticals; spiked human serum	[27]
MoSe_2_/rGO/β-CyD/GCE	DPV	1.90 × 10^−7^–3.75 × 10^−4^	2.80 × 10^−8^	Human serum; urine; river water; fish	[37]
PVP capped CoFe_2_O_4_@CdSe/GCE	AdSSWV	1.00 × 10^−16^–1.00 × 10^−7^	4.55 × 10^−17^	Pharmaceuticals; human serum	[112]
PMel-AuNPs/GCE*	LSV	8.00 × 10^−8^–1.50 × 10^−5^	3.00 × 10^−8^	Spiked human urine	[45]
MIPPy/Cu-MOF/MC/GCE	AdSDPV	8.00 × 10^−8^–8.50 × 10^−5^	2.80 × 10^−10^	Pharmaceuticals; human serum; urine	[68]
ZrO_2_@CS/GCE	CV	1.50 × 10^−8^–5.47 × 10^−4^	7.50 × 10^−9^	Human serum; urine	[113]
CS/Au/VXC72R/GCE	Amperometry	5.00 × 10^−7^–1.00 × 10^−5^	1.10 × 10^−7^	Bovine serum	[114]
BiVO_4_/SPCE	CV; LSV	2.00 × 10^−7^–3.10 × 10^−4^	1.40 × 10^−8^	Human serum; urine	[53]
F_64_PcZn/TiO_2_/SPE	PEC	Layer: 1.00 µg/mm^2^ 5.00 × 10^−8^–2.50 × 10^−6^ Layer: 4.00 µg/mm^2^ 1.00 × 10^−7^–1.00 × 10^−5^	7.00 × 10^−9^ 2.80 × 10^−8^	Waste water	[115]
Electroactivated SPBDDE	AdSDPV	2.00 × 10^−12^–1.00 × 10^−11^ 2.00 × 10^−11^–2.00 × 10^−10^ 2.00 × 10^−10^–2.00 × 10^−9^ 2.00 × 10^−9^–2.00 × 10^−8^	2.20 × 10^−13^	River water; bovine urine (certified reference material).	[36]
PGE	AdSDPV	1.99 × 10^−8^–1.20 × 10^−7^	1.30 × 10^−8^	Pharmaceuticals; urine, spiked human serum	[84]
CPE	SWV AdSSWV	5.00 × 10^−7^–5.00 × 10^−5^ 1.00 × 10^−7^–6.00 × 10^−6^	2.35 × 10^−7^ 1.72 × 10^−8^	Pharmaceuticals; spiked human serum	[80]
DyNW/CPE	AdSSWV	1.00 × 10^−10^–1.00 × 10^−7^	5.00 × 10^−10^	Pharmaceuticals; spiked human serum	[116]
HP-β-CyD/CPE	Potentiometry	3.20 × 10^−8^–2.20 × 10^−4^	2.30 × 10^−8^	Pharmaceuticals; human blood serum	[21]
CDs@CuFe_2_O_4_/CPE	-	7.00 × 10^−8^–8.00 × 10^−6^	2.20 × 10^−8^	Biological fluids; pharmaceuticals	[88]
CPE in situ SDS modified CPE	AdSDPV_anodic_ AdSDPV_cathodic_ AdSDPV_anodic_ AdSDPV_cathodic_	3.50 × 10^−10^–5.40 × 10^−9^ 9.00 × 10^−11^–2.90 × 10^−9^ 3.50 × 10^−10^–5.40 × 10^−9^ 9.00 × 10^−11^–1.80 × 10^−9^	-	-	[26]
Mn_3_O_4_@SiO_2_/CPME	SWV	3.00 × 10^−8^–3.00 × 10^−6^	1.08 × 10^−8^	Spiked human serum; urine	[117]
NiTAPc-GO/ITO	PEC	2.50 × 10^−8^–7.13 × 10^−5^	2.50 × 10^−9^	Pharmaceuticals	[118]
EG-CYP2E1/PVP-AgNPs/PANSA/Au	DPV	2.00 × 10^−6^–1.40 × 10^−5^	5.00 × 10^−8^	Human serum	[50]
CYP3A4/CuPPI/Au	DPV	2.00 × 10^−10^–1.00 × 10^−9^	1.07 × 10^−10^	Spiked synthetic plasma and urine	[86]
HRP/PPy/Pt	Amperometry/ H_2_O_2_	Chemometrics	5.00 × 10^−6^	Pharmaceuticals; urine	[119]
PPy-β-CyD/Pt	Amperometry	2.61 × 10^−6^–2.52 × 10^−5^	1.69 × 10^−6^	Pharmaceuticals; urine	[120]
Rifamycin SV (RSV)					
HMDE	AdSDPV AdSSWV	Chemometrics	3.11 × 10^−8^ 1.23 × 10^−8^	Pharmaceuticals	[105]
PGE	AdSDPV	1.90 × 10^−8^–4.10 × 10^−7^	6.00 × 10^−8^	Pharmaceuticals; urine, spiked human serum	[84]
MI polyphosphazenes/GCE	DPV	2.56 × 10^−7^–6.36 × 10^−6^	3.99 × 10^−5^	-	[121]
CPE in situ CTAC modified CPE	AdSDPV_anodic_ AdSDPV_cathodic_ AdSDPV_anodic_ AdSDPV_cathodic_	5.00 × 10^−11^–1.00 × 10^−9^ 3.00 × 10^−11^–8.30 × 10^−10^ 5.00 × 10^−11^–1.00 × 10^−9^ 9.00 × 10^−11^–6.20 × 10^−9^	-	-	[26]

**Table 2:** The performance characteristics of spectrometric methods reported in the literature for rifamycins determination.

Analyte	Method	Wavelength (nm)	LR (µg/mL)	LOD (µg/mL)	Sample	Ref.
RIF	direct water	474	0.82–65.38	-	Pharmaceuticals; urine, plasma	[138]
RIF	HCl H_3_PO_4_	263 259	1.50–30.00	0.19 0.14	Pharmaceuticals; urine	[141]
RIF	PBS pH 7.00	470	8.00–128.00	0.16	-	[142]
RIF	direct ethyl acetate	334	2.50–35.00	0.83	Combined dosage forms	[136]
RIF ISN	AA water	337 263	5.00–35.00 5.00–25.00	1.65 0.59	Combined dosage forms	[125]
RIF ISN RIF ISN	AA methanol 1D methanol	338 263 263 290	5.00–50.00	3.50 2.60 2.30 1.30	Combined dosage forms	[126]
RIF PYR	1D methanol	365 247	4.00–12.00	0.87 0.82	-	[127]
RIF Piperine	Q-AR methanol	387 337	5.00–40.00 2.00–20.00	1.51 0.28	Combined dosage forms	[128]
RIF CC-I	Q-AR methanol:water	370 239	2.00–20.00 1.00–24.00	0.043 0.014	In-house combined formulation	[129]
RIF PYR ISN RIF PYR ISN RIF PYR ISN	DRSZ DDRD HDDR	358 252 294 350 259 293 292 324 & 345 279 & 286	5.00–30.00 5.00–30.00 5.00–30.00 2.00–30.00 2.00–30.00	2.14 1.39 1.76 1.76 1.62 1.8 0.64 0.43 1.61	Pharmaceuticals; urine	[130]
RIF	Chloranilic acid	510	7.90–39.10	-	Pharmaceuticals	[143]
RIF	DDQ TCNQ TCNQ *p-*chloranil Fe (III)	584 680 761 560 540	5.00–140.00 5.00–120.00 2.00–45.00 15.00–200.00 10.00–240.00	2.59 2.09 0.90 3.95 2.30	Pharmaceuticals	[139]
RIF	FCR Indirect/Fe(III) + K_3_[Fe(CN)_6_]	760 750	1.00–35.00 2.50–50.00	0.32 0.32	Pharmaceuticals; urine	[140]
RIF	Indirect/NBS + KI	572	0.50–15.50	-	Pharmaceuticals; urine; plasma	[138]
RIF ISN	Cu(II) + neocuproine/ PLS regression		8.00–57.00 5.50–7.00	0.06 0.04	Combined dosage forms; urine	[132]

**Table 3:** The performance characteristics of the analytical methods based on fluorescence sensors reported in the literature for rifamycins determination.

Analyte	Sensor	Wavelength (nm)		LR (mol/L)	LOD (mol/L)	Sample	Ref.
		Excitation	Emission				
RIF	(GSH)-capped CdTe/ZnS QDs	350	577	1.00 × 10^−6^–6.80 × 10^−5^	3.04 × 10^−7^	Capsules	[148]
RIF	CatSt-GSH-capped CdTe/ZnS QDs	475	576	4.05 × 10^−6^–3.65 × 10^−5^	6.00 × 10^−8^	Capsules	[149]
RIF	CDs-HSs	400	480	2.40 × 10^−6^–3.38 × 10^−5^	4.60 × 10^−7^	Lake and tap water	[155]
RIF	N-P-CNDs	340	450	1.00 × 10^−6^–1.00 × 10^−4^	6.00 × 10^−8^	Capsules	[97]
RIF	PEI-capped Cu NCs	362	492	0.00–2.00 × 10^−5^	5.00 × 10^−9^	Human serum	[151]
RIF	GSH-Cu NCs	-	632	5.00 × 10^−11^–1.00 × 10^−8^	1.60 × 10^−11^	-	[153]
RIF	FA capped Cu NCs	358	446	5.00 × 10^−7^–1.00 × 10^−4^	7.30 × 10^−8^	Capsules; bovine serum; milk	[152]
RIF	UCCS NaYF_4_:Yb, Tm@SiO_2_	Laser 980	475	0.00–2.00 × 10^−4^	8.50 × 10^−6^	Human urine	[154]
RFPT RIF RFD RSV	BSA	285	355	8.00 × 10^−9^–5.00 × 10^−5^ 9.00 × 10^−9^–5.00 × 10^−5^ 1.60 × 10^−8^–4.00 × 10^−5^ 1.80 × 10^−8^–4.00 × 10^−5^	7.60 × 10^−10^ 8.90 × 10^−10^ 1.55 × 10^−9^ 1.77 × 10^−9^	Capsules; human urine	[30]
RFPT RIF RFD RSV	HSA			1.00 × 10^−8^–4.00 × 10^−5^ 1.10 × 10^−8^–4.00 × 10^−5^ 1.90 × 10^−8^–3.50 × 10^−5^ 1.90 × 10^−8^–3.50 × 10^−5^	8.50 × 10^−10^ 9.80 × 10^−10^ 1.83 × 10^−9^ 1.89 × 10^−9^		
RIF	BSA-Au NCs	480	640	6.07 × 10^−7^–1.00 × 10^−3^	8.50 × 10^−8^	Human urine	[54]

**Table 4:** The performance characteristics of the analytical methods based on CL sensors reported in the literature for RIF analysis.

Reagent/Sensor	Method	LR (mol/L)	LOD (mol/L)	Sample	Ref.
KHSO_5_ + CoSO_4_	FI-CL	6.08 × 10^−8^–1.22 × 10^−6^	8.50 × 10^−9^	Capsules; eye drops	[49]
NBS in NaOH + NH_3_	CF-CL	1.22 × 10^−8^–1.22 × 10^−^	6.07 × 10^−9^	Combined pharmaceuticals	[81]
B,N-doped CDs Ce(SO_4_)_2_ + Na_2_SO_3_	FI-CL	2.00 × 10^−10^–1.5 × 10^−7^	5.00× 10^−11^	Spiked plasma; spiked urine	[44]
K_2_S_2_O_8_/Pt coil electrode	FI-ECL	1.00 × 10^−7^–4.00 × 10^−5^	3.90 × 10^−8^	Pharmaceuticals; urine	[40]
Luminol/Pt flake electrode	ECL	1.00 × 10^−8^–4.00 × 10^−6^	8.00 × 10^−9^	Pharmaceuticals; urine	[157]

## Data Availability

Not applicable.

## Abbreviations

1D	first derivative
AA	absorbancies additivity
ABS	acetate buffer solution
AdSDPV	adsorptive stripping differential pulse voltammetry;
AdSSWV	adsorptive stripping square wave voltammetry;
ATR-FTIR	attenuated total reflectance Fourier-transform infrared spectroscopy
BSA	bovine serum albumin
C120	coumarin 120
CC-I	3′,5-dihydroxyflavone-7-O-β-D-galacturonide-4′-O-β-D-glucopyranoside from *Cuminum cyminum* seeds
CatSt	cationic starch
CDs	carbon dots
CDs-HSs	carbon dots hydrogel spheres
CF	continuous flow
CL	chemiluminescence
CLS	classical least square
CoEnQ10	coenzyme Q10
CoTHPP	*meso*-tetrakis(4-hydroxyphenyl)porphyrinato cobalt(II)
CP(E)	carbon paste (electrode)
CP(ME)	carbon paste (microelectrode)
CS	chitosan
CSWV	cyclic square-wave voltammetry
CTAC	cetyltrimethylammonium chloride
CV	cyclic voltammetry
CyD	cyclodextrin
CYP3A4/CuPPI	cytochrome P450 3A4 attached to copper polypropyleneimine
DACCA	7-(diethylamino)-coumarin-3-carboxylic acid N-succinimidyl ester
DDRD	double divisor ratio spectra derivative
DRSZ	derivative ratio spectra-zero-crossing
DDQ	2,3-dichloro-5,6-dicyano-1,4-benzoquinone
DME	dropping mercury electrode
DMF	dimethylformamide
DMSO	dimethylsulfoxide
DPP/DPV	differential pulse polarography/differential pulse voltammetry
ds	double stranded
DyNW	dysprosium nanowires
ECL	electrochemiluminescence
EG-CYP2EI	ethylene glycol bis(succinic acid N-hydroxysuccinimide ester)-modified cytochrome P450 2E1
EIS	electrochemical impedance spectroscopy
ETB	ethambutol
F_64_PcZn/TiO_2_/SPE	perfluorinated phthalocyanine zinc complex deposited on TiO_2_/screen printed electrode
FA	folic acid
FCR	Folin-Ciocalteu reduction
FI	flow injection
FRET	Förster resonance energy transfer
FTIR	Fourier-transform infrared spectroscopy
GC(E)	glassy carbon (electrode)
GCE*	pre-anodized GCE
GMO	gadolinium manganese oxide
GNPls	graphene nanoplatelets
GO	graphene oxide
GSH	glutathione
HDDR	hybrid double divisor ratio spectra
HgFE	mercury film electrode
HIV	human immunodeficiency virus
HMDE	hanging mercury drop electrode
HP-β-CyD	2-hydroxypropyl β-cyclodextrin
HPLC-ECD	high performance liquid chromatography-electrochemical detection
HRP	horseradish peroxidase
HSA	human serum albumin
IR	infrared
ISN	isoniazide
ITO	indium tin oxide
LOD	limit of detection
LOQ	limit of quantification
LR	linear range
LSV	linear sweep voltammetry
MEC	microelectrodes modified with Chitosan
MI(Ps)	molecular imprinted (polymers)
MIPPy-Cu-MOF/MC	molecularly imprinted polypyrrole/copper metal organic framework/mesoporous carbon
MSNPs	mesoporous silica nanoparticles
Mtb	mycobacteria tuberculosis
MWCNTs	multi-walled carbon nanotubes
NBS	N-bromosuccinimide
NCs	nanoclusters
NiHCF	nickel hexacyanoferrate
NIR	near infrared
N-P-CNDs	nitrogen and phosphorous-doped carbon nanodots
NPs	nanoparticles
NRIF	nanosome rifampicin (RIF encapsulated in phospholipid micelles)
NRs	nanorods
NSs	nanosheets
NiTAPc	nickel tetraamino phthalocyanine
OFM	orthogonal function method
PB_290_-*b*-PDMAEMA_240_	poly(1,2-butadiene)-*block*- poly(2-(dimethylamino)ethyl methacrylate (the subscripts denote the number-average degrees of polymerization of the respective segment)
PBS	phosphate buffer solution
PCR	principale component regression
PEC	photoelectrochemistry
PEG	polyethylene glycol
PEI	polyethyleneimine
PG(E)	pencil graphite (electrode)
PLS	partial least squares
PMel	polymelamine
PPy	polypyrrole
PVP	polyvinyl pyrrolidone
PANSA	poly(8-anilino-1-naphthalene sulphonic acid)
PYR	pyrazinamide
Q-AR	Q-absorption ratio
QDs	quantum dots
rGO	reduced graphene oxide
RFPT	rifapentine
RFD	rifandin
RIF	rifampicin
RSV	rifamycin SV
SCV	small colony variants
SDS	sodium dodecylsulphate
seATRP	simplified electrochemically mediated atom transfer radical polymerization
SMDE	static mercury drop electrode
SPBDDE	screen-printed boron-doped diamond electrode
SPCE	screen-printed carbon electrode
SPE	screen-printed electrode
SPION	superparamagnetic iron oxide nanoparticles
ss	single stranded
SWP / SWV	square-wave polarography/square-wave voltammetry
TB	tuberculosis
TCNQ	7,7,7,8-tetracyano quinodimethane
TDM	therapeutic drug monitoring
TiO_2_/rGO	titanium dioxide nanoparticles anchored reduced graphene oxide sheets
UCCS	upconversion core-shell
UV	ultraviolet
Vis	visible
VXC72R	Cabot Vulcan XC72R carbon black
WHO	world health organization
